# Supplementary material for: Sedation Quality and Cardiorespiratory, Echocardiographic, Radiographic and Electrocardiographic Effects of Intramuscular Alfaxalone and Butorphanol in Spanish Greyhound Dogs
Source: Animals (Basel). 2023 Sep 16;13(18):2937. doi: 10.3390/ani13182937 (PMC10525580; doi:10.3390/ani13182937)
Supplement: Supplementary file 1 [file animals-13-02937-s001.zip › animals-2540682-supplementary.pdf]

Supplementary Materials:

Scale A	
Score 0 (Normal)	No visible signs of sedation
Score 1 (Mild)	Decreased alertness, some response to acoustic stimuli (hand clap)<br>Head down, strong palpebral reflex, normal eye position
Score 2 (Moderate)	Lateral or sternal recumbency, minimal response to acoustic stimuli<br>Head down, moderate palpebral reflex, partial ventromedial eye rotation
Score 3 (Deep)	Lateral or sternal recumbency, no response to acoustic stimuli<br>Head down, weak palpebral reflex, complete ventromedial eye rotation

Scale B	
Variable	Score: Description
Posture	(0) Standing, normal proprioception and no ataxia<br>(1) Animal remains in sternal or lateral position; able to stand when stimulated verbally<br>(2) Remains in sternal recumbency<br>(3) Lateral recumbency; eventually lifts or moves head<br>(4) Lateral recumbency; if not verbally stimulated, does not move or lift its head
Response to sound	(0) Alert attitude; readily reacts (looks, lifts or moves head) to the stimulus<br>(1) Reduced reaction (discrete movement, lifting of the head), but the animal appears sedated<br>(2) No reaction or movement
Resistance to physical restraint in lateral recumbency	(0) Animal resists; readily returns to standing position or sternal recumbency after being released<br>(1) Offers little resistance, but readily returns to standing position or sternal recumbency<br>(2) Does not offer resistance, but eventually moves or lifts head and returns to sternal recumbency<br>(3) Remains in lateral recumbency; does not offer resistance
General appearance	(0) Alert, normal consciousness<br>(1) Animal lightly sedated; promptly reacts or moves in response to environmental stimulus<br>(2) Animal moderately sedated; eventually reacts to environmental stimulus<br>(3) Animal appears moderately to deeply sedated; reduced reaction to environmental stimulation<br>(4) Animal appears to be deeply sedated; does not react to environmental stimulation

* Total sedation score was assigned as a sum of scores for each variable. Bright, alert, responsive dogs would have received a score of 0. The maximum score was 13 and indicated the deepest level of sedation.

Scale C	
Parameter	Response
Spontaneous position	(0) Able to stand and walk<br>(1) Sedated but standing or sitting<br>(2) Lying down but able to react quickly or stand up<br>(3) Lying down, but reacting slowly and having difficulty in standing up<br>(4) Lying down and unable to stand up
Resistance to lateral recumbency	(0) Strong resistance<br>(1) Moderate resistance<br>(2) Slight resistance<br>(3) No resistance
Response to noise	(0) Normal response<br>(1) Listens and moves<br>(2) Listens and ear moves<br>(3) Hardly perceives<br>(4) No response
Jaw relaxation	(0) Normal<br>(1) Slightly reduced<br>(2) Greatly reduced
Eyelid reflex	(0) Normal<br>(1) Depressed reflex<br>(2) No reflex
Response to pain	(0) Normal response (withdrawal of the limb at a minimal clamping pressure)<br>(1) Slow response (withdrawal at a higher clamping pressure)<br>(2) Very slow response (withdrawal at a higher clamping pressure maintained for 3 to 5-seconds)<br>(3) No response

* The sedation was judged to be poor (total score 0–3), mild (total score 4–6), moderate (total score 7–10) and deep (total score 11–15). Total scores > 15 were considered as an anaesthesia state

* Modified numerical rating scale (Young et al. 1990) (range from 0 = low value to 4 = high value) of six independent parameters used for scoring the degree of sedation and anaesthesia after the intramuscular (IM) administration of alfaxalone alone or in combination with dexmedetomidine in cats. The sedation was judged to be poor (total score 0–3), mild (total score 4–6), moderate (total score 7–10) and deep (total score 11–15). Total scores > 15 for intubated cats were considered as an anaesthesia state.
